# Ginsenoside Rg3 inhibits epithelial-mesenchymal transition (EMT) and invasion of lung cancer by down-regulating FUT4

**DOI:** 10.18632/oncotarget.6451

**Published:** 2015-12-02

**Authors:** Lili Tian, Dachuan Shen, Xiaodong Li, Xiu Shan, Xiaoqi Wang, Qiu Yan, Jiwei Liu

**Affiliations:** ^1^ Department of Oncology, First Affiliated Hospital of Dalian Medical University, Dalian, Liaoning Province, China; ^2^ Institute of Cancer Stem Cell, Dalian Medical University, Dalian, Liaoning Province, China; ^3^ Department of Dermatology, Northwestern University Feinberg School of Medicine, Chicago, Illinois, United States of America; ^4^ Department of Biochemistry and Molecular Biology, Liaoning Provincial Core Lab of Glycobiology and Glycoengineering, Dalian Medical University, Dalian, Liaoning Province, China

**Keywords:** Rg3, EMT, FUT4, EGFR, lung cancer

## Abstract

The epithelial-mesenchymal transition (EMT) is an important factor in lung cancer metastasis, and targeting EMT is a potential therapeutic strategy. Fucosyltransferase IV (FUT4) and its synthetic cancer sugar antigen Lewis Y (LeY) was abnormally elevated in many cancers. In this study, a traditional Chinese medicine ginsenoside Rg3 was used to investigate whether its inhibition to EMT and invasion of lung cancer is by the glycobiology mechanism. We found that Rg3 treatment (25, 50, 100 μg/ml) inhibited cell migration and invasion by wound-healing and transwell assays. Rg3 could significantly alter EMT marker proteins with increased E-cadherin, but decreased Snail, N-cadherin and Vimentin expression. Rg3 also down-regulated FUT4 gene and protein expression in lung cancer cells by qPCR, Western blot and immunofluorescence. After FUT4 down-regulated with shFUT4, EMT was obviously inhibited. Furthermore, the activation of EGFR through decreased LeY biosynthesis was inhibited, which blocked the downstream MAPK and NF-κB signal pathways. In addition, Rg3 reduced tumor volume and weight in xenograft mouse model, and significantly decreased tumor metastasis nodules in lung tissues by tail vein injection. In conclusion, Rg3 inhibits EMT and invasion of lung cancer by down-regulating FUT4 mediated EGFR inactivation and blocking MAPK and NF-κB signal pathways. Rg3 may be a potentially effective agent for the treatment of lung cancer.

## INSTRUCTION

Lung cancer is the most common malignancy worldwide, and it ranks first in cancer mortality [1]. Approximately 300,000 people are diagnosed with lung cancer in China every year, and more than 250,000 patients die from the disease [2]. There are generally two types of lung cancer: small-cell lung cancer and non-small cell lung cancer (NSCLC). NSCLC is the most common type, accounting for nearly 85 % of cases [3]. Although chemotherapy has made great progress in the last few decades, and targeted drugs have been developed in recent years, the side effects and drug resistance cause most patients die of cancer invasion and metastasis, with a 5-year survival rate of less than 15 % [4]. Invasion and metastasis is the leading factor that result in the failure of lung cancer treatment and poor prognosis. Therefore, there is an urgent need to seek anti-metastatic drugs with explicit mechanisms and explore new targets for lung cancer.

The epithelial mesenchymal transition (EMT) plays an important role in the migration, invasion and metastasis of tumors. Moreover, EMT shortens progression free survival (PFS) and overall survival (OS) of the patients [5]. A large number of molecular markers and pathways correlated with EMT have been explored. In the EMT process, EMT features a series of biological events, including the decreased expression of the epithelial marker E-cadherin protein, leading to a lost connection between epithelial cells and decreased polarity of the epithelial cells. However, the expression of N-cadherin and Vimentin is increased, which exhibits a variety of mesenchymal cell properties, and enhances migration and invasion potential [6]. The EMT process is regulated by a set of transcription factors, including Slug, Snail, Twist, ZEB1 and ZEB2. These transcription factors transform epithelial state into the mesenchymal state by inhibiting the epithelial marker proteins expression and inducing the mesenchymal state-related marker proteins expression [7]. Induced EMT has significantly promoted the metastasis of ovarian cancer, breast cancer and osteosarcoma by activating some signal pathways, such as TGF-β MAPK and NF-κB, and these signal pathways affect each other interactively [8, 9]. EMT can also increase drug resistance for radiation and chemotherapy [10]. Drugs inhibiting EMT may be effective agents for lung cancer.

Tumor glycobiology studies indicate that alterations in the fucosylation of cancer cells closely correlates with tumor growth and metastasis, et al. Abnormal fucosylation is catalyzed by the specific fucosyltransferases (fucosyltransferases, FUTs). FUT4 catalyzes the transfer of the Fuc of GDP-Fuc to the N-acetylglucosamine of sugar chain, and it is a key enzyme in the synthesis of tumor associated carbohydrate antigen Lewis Y (LeY) [11]. LeY induces the activation of EGFR, and promotes the migration of oral cancer cells by the glycosylation of EGFR [12,13]. FUT4 is highly expressed in a variety of cancers, including stomach cancer, pancreatic cancer, colon cancer and ovarian cancer, is closely correlated to tumor proliferation, apoptosis, metastasis and EMT. However, whether down-regulation of FUT4 can inhibit EMT in lung cancer is still unclear.

Ginsenosides are active pharmaceutical ingredients extracted from the traditional Chinese medicine ginseng. Currently, 40 types of ginsenoside compounds have been identified, among which, Rg3 is attracting increasing attention. Rg3 has multiple antitumor effects. Rg3 inhibits the proliferation of colorectal cancer by inhibiting the Wnt/Δ-catenin pathway [14], promotes apoptosis of ovarian cancer cells by inhibiting the PI3K/AKT pathway [15], inhibits angiogenesis of esophageal cancer induced by oxygen deficit [16], and inhibits EMT in ovarian cancer by reducing HIF-1α [17]. Rg3 combined with gemcitabine or As_2_O_3_ decreased the angiogenesis and growth of lung cancer in mice, respectively [18,19]. Recently, it is reported that Rg3 could inhibit TGF-β1-induced EMT in lung cancer, as well as cancer migration and invasion [20], indicating that Rg3 may be a potential therapeutic agent that targets EMT. However, whether Rg3 inhibits lung cancer EMT by down-regulating FUT4 mediated glycosylation is not clear. In this study, for the first time, we found that Rg3 effectively inhibited migration, invasion, EMT and metastasis by down-regulating FUT4 in human lung cancer *in vitro* and *in vivo*. Therefore, our results provide new insights, suggesting that Rg3 may be a novel anti-metastasis agent to treat lung cancer.

## RESULTS

### Rg3 inhibited the migration and invasion of human NSCLC cells

**Figure 1:**
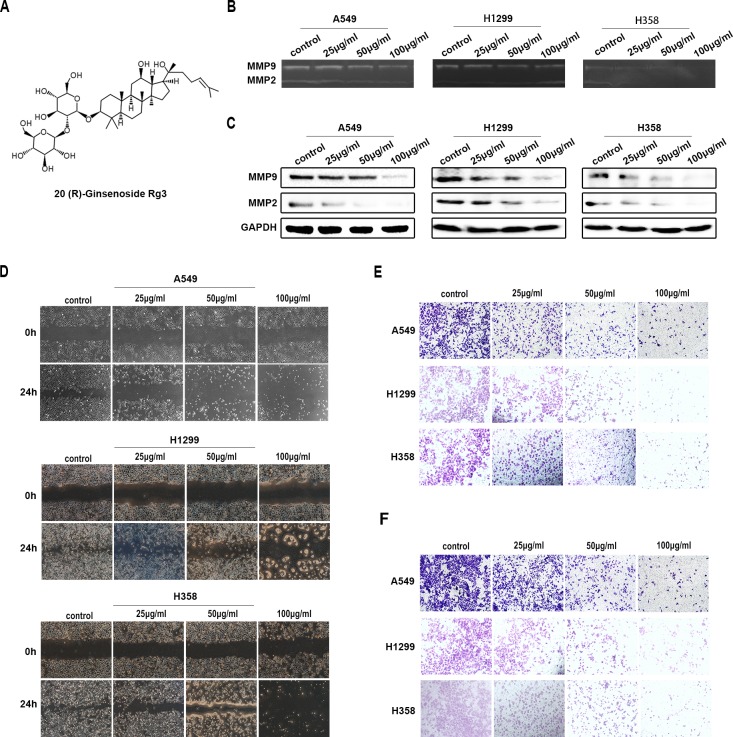
Rg3 inhibited migration and invasion of human NSCLC cells **A.** Structure of 20 (R)-Ginsenoside Rg3. A549, H1299 and H358 cells were treated with Rg3 (0, 25, 50, 100 μg/ml) for 24 h. **B.** Analysis of MMP2 and MMP9 activity by gelatin zymography. **C.** MMP2 and MMP9 expression by Western blot. GAPDH was used as an internal control. Cell migration was detected by wound-healing assay **D.** and transwell assay with non-coated membrane **E. F.** Cell invasion was examined by transwell assay with Matrigel-coated membrane.

The structure of Rg3 is shown in Figure 1A. To evaluate the effects of Rg3 on inhibiting the migration and invasion of human NSCLC cells, we treated A549, H1299 and H358 cells with different concentration of Rg3 (0, 25, 50, 100 μg/ml) for 24 h. The gelatin zymography assay showed that the activities of MMP2 and MMP9 were degraded (Figure 1B). Western blot showed that MMP2 and MMP9 expression was reduced (Figure 1C). Cell migration was evaluated by wound-healing and transwell assay with non-coated membrane. We found that Rg3 clearly inhibited wound closure (Figure 1D) and the cellular migration through the membrane (Figure 1E). To detect the alteration in the invasive capability after Rg3 treatment, we used transwell assay by Matrigel precoated on the membrane, and got parallel results (Figure 1F). The above studies also showed a dose-dependent manner with Rg3 treatment. These data suggest that Rg3 can effectively inhibit the migration and invasion of human NSCLC cells.

### Rg3 inhibited EMT of human NSCLC cells

**Figure 2:**
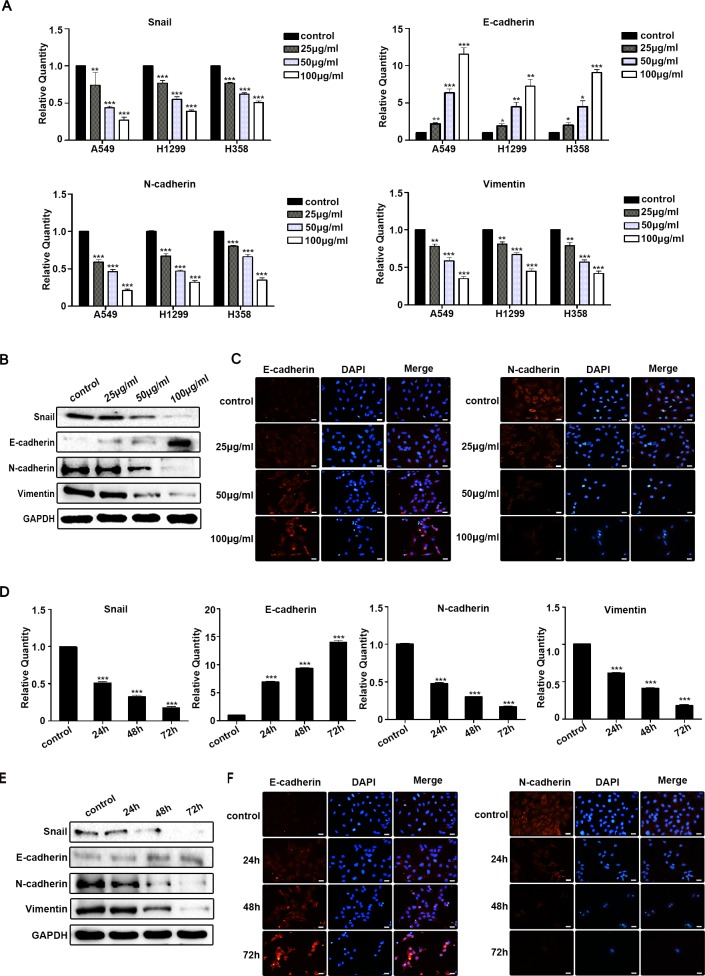
Rg3 inhibited EMT of human NSCLC cells **A.** Snail, E-cadherin, N-cadherin and Vimentin were detected by qPCR in A549, H1299 and H358 cells after treatment with Rg3 (0, 25, 50, 100 μg/ml) for 48 h. Snail, E-cadherin, N-cadherin and Vimentin were detected by Western blot **B.** and immunofluorescent staining of E-cadherin and N-cadherin **C.** in A549 cells after Rg3 treatment for 24 h. Snail, E-cadherin, N-cadherin and Vimentin were detected by qPCR **D.**, Western blot **E.** and immunofluorescent staining of E-cadherin and N-cadherin **F.** in A549 cells after treatment with Rg3 (50 μg/ml) for 0, 24, 48 or 72 h. GAPDH was used as an internal control. DAPI was used for nuclear staining (bar = 50 μm; magnification, 400x). The statistical analysis of qPCR is shown (*, *P* < 0.05; **, *P* < 0.01; ***, *P* < 0.001). The data are presented as the mean ± SEM of three independent experiments.

We next examined the ability of Rg3 to inhibit EMT in human NSCLC cells. A549, H1299 and H358 cells were treated with different concentration of Rg3 (0, 25, 50, 100 μg/ml) for 48 h. The results indicated a significant up-regulation of the E-cadherin and down-regulation of Snail, N-cadherin and Vimentin by qPCR (Figure 2A). The inhibitory effects of Rg3 on the EMT markers were further confirmed by Western blot in A549 cells treated differently with Rg3 for 24 h (Figure 2B). We also found that E-cadherin staining was enhanced; whereas N-cadherin staining weakened by immunofluorescent analysis in A549 cells (Figure 2C). Treating A549 cells with Rg3 at 50 μg/ml for 0, 24, 48, or 72 h, the results were consistent with an elevated level of E-cadherin and a reduced level of Snail, N-cadherin and Vimentin by qPCR (Figure 2D), Western blot (Figure 2E) and immunofluorescent staining (Figure 2F). These data suggest that Rg3 effectively prevent EMT in a dose- and time-dependent manner in NSCLC cells.

### Rg3 decreased EMT by down-regulating FUT4 in lung cancer cells

**Figure 3:**
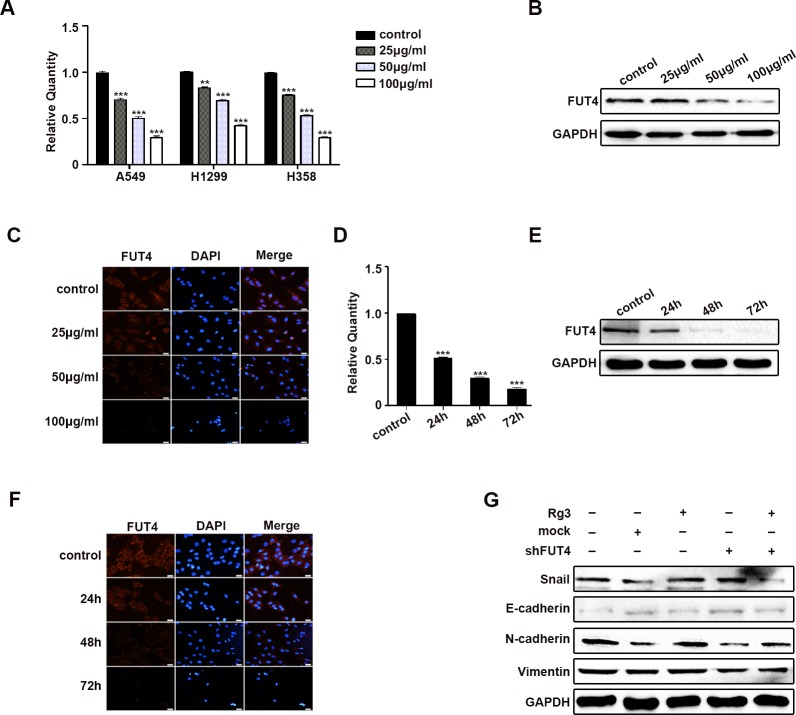
Rg3 decreased EMT by down-regulating FUT4 in lung cancer cells A549 cells treated with Rg3 (0, 25, 50, 100 μg/ml) for 48 h were collected. FUT4 expression was detected by qPCR **A.**, Western blot **B.** and immunofluorescent staining **C.** A549 cells treated with Rg3 (50 μg/ml) for 0, 24, 48 or 72 h were collected. FUT4 expression was detected by qPCR **D.**, Western blot **E.** and immunofluorescent staining **F.**. A549 cells were treated with Rg3 (50 μg/ml), FUT4 shRNA, and FUT4 shRNA transfection followed by Rg3 treatment. Snail, E-cadherin, N-cadherin and Vimentin expression were detected by Western blot **G.**. Control, untreated cells; Mock, cells transfected with vector. GAPDH was used as an internal control. DAPI was used for nuclear staining (bar = 50 μm; magnification, 400x). The statistical analysis of qPCR is shown (**, *P* < 0.01; ***, *P* < 0.001). The data are presented as the mean ± SEM of three independent experiments.

To elucidate the mechanism by which Rg3 reduced EMT, FUT4 expression was examined in human normal lung and lung cancer paraffin sections. Representative FUT4 staining using immunohistochemistry (IHC) was shown in Figure S1A. The positive FUT4 expression rate was 11.4 % (4/35) in normal lung tissues, and 60.9 % (39/56) in lung cancer tissues (Figure S1B, *P* < 0.001). To further confirm FUT4 expression was high in lung cancer, Western blot was used to analyze the 10 paired normal lung and lung cancer tissues. A representative picture of the results was shown in Figure S1C. FUT4 expression in lung cancer tissues was higher than that in normal lung tissues (Figure S1D, *P* < 0.001). We treated A549, H1299 and H358 cells with different concentration of Rg3 (0, 25, 50, 100 μg/ml) for 48 h, and the results showed that FUT4 expression was suppressed by qPCR (Figure 3A). The changes of FUT4 protein in A549 cells after Rg3 treatment was further analyzed by Western blot (Figure 3B) and immunofluorescent staining (Figure 3C), and the results showed that FUT4 expression was down-regulated.

After treating A549 cells with Rg3 at 50 μg/ml for 0, 24, 48, or 72 h, the results showed that FUT4 expression was reduced by qPCR (Figure 3D), Western blot (Figure 3E) and immunofluorescent staining (Figure 3F). Therefore, Rg3 effectively down-regulated expression of FUT4 in a dose- and time-dependent manner. After Rg3 treatment, shFUT4 infection, or Rg3 treatment in combination with shFUT4 infection in A549 cells, the expression of EMT marker proteins present a similar tendency (Figure 3G) as mentioned above. Thus, these results suggest that Rg3 plays an important role in inhibiting EMT by down-regulating FUT4 in NSCLC cells.

### Down-regulating FUT4 expression reduced migration, invasion and EMT in A549 cells

**Figure 4:**
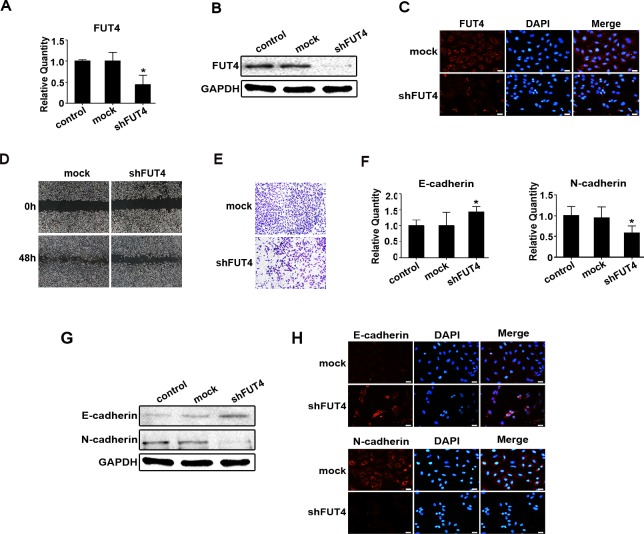
Down-regulating FUT4 expression reduced migration, invasion and EMT in A549 cells A549 cells were transfected with FUT4 shRNA. FUT4 expression was detected by qPCR **A.**, Western blot **B.** and immunofluorescent staining **C. D.** Cell migration was detected by wound-healing assay. **E.** Cell invasion was examined by transwell (Matrigel-coated membrane). E-cadherin and N-cadherin were detected by qPCR **F.**, Western blot **G.** and immunofluorescent staining **H.**. Control, untransfected cells; Mock, cells transfected with vector; shFUT4, cells transfected with FUT4 shRNA. GAPDH was used as an internal control. DAPI was used for nuclear staining (bar = 50 μm; magnification, 400x). The statistical analysis of qPCR is shown (*, *P* < 0.05). The data are presented as the mean ± SEM of three independent experiments.

To investigate whether down-regulating FUT4 expression inhibited migration, invasion and EMT in lung cancer, we analyzed the potential correlation between FUT4 and EMT in lung cancer tissues. We collected paraffin sections to examine FUT4 and N-cadherin protein expression, the results showed FUT4 and N-cadherin were more highly expressed in lung cancer than normal lung tissues (Figure S2A), and they had the same tendency. We used Western blot to analyze FUT4 and N-cadherin protein expression in fresh lung cancer tissues. The results showed that FUT4 was positively correlated with N-cadherin (Figure S2B, C, *P* < 0.05). We also developed shRNA interference sequences (shFUT4) to silence FUT4 expression in A549 cells. As showed in Figure 4A, 4B, 4C, FUT4 expression was suppressed by shFUT4 compared with the untreated control and mock transfected cells. Cell migration and invasion were evaluated in wound-healing and transwell assays. Wound closure (Figure 4D) and invasion (Figure 4E) were significantly inhibited in shFUT4 transfected cells. Furthermore, we examined EMT marker proteins, and found that E-cadherin expression was increased and N-cadherin expression was decreased by qPCR (Figure 4F), Western blot (Figure 4G) and immunofluorescent staining (Figure 4H) in shFUT4 transfected cells compared with the untreated control and mock transfected cells. These results suggest that down-regulating FUT4 expression can inhibit migration, invasion and EMT in lung cancer cells.

### Down-regulating FUT4 expression inhibited LeY biosynthesis, EGFR activation, MAPK and NF-κB signal pathways in A549 cells

**Figure 5:**
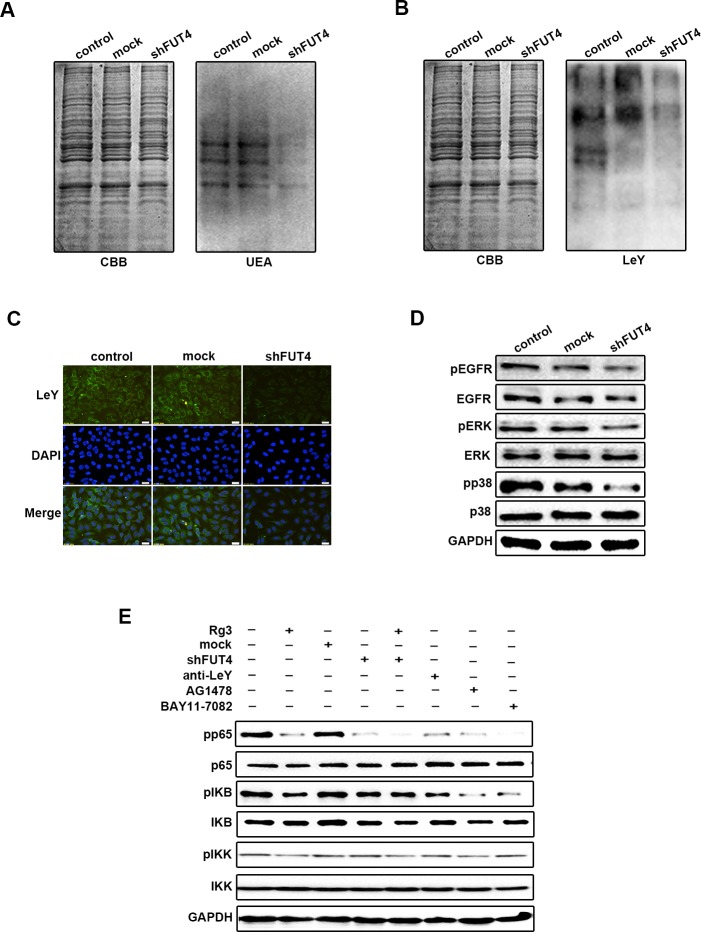
Down-regulating FUT4 expression inhibited LeY biosynthesis, EGFR activation, MAPK and NF-κB signal pathways in A549 cells A549 cells transfected with FUT4 shRNA were collected. UEA **A.** and LeY **B.** expression were detected by Western blot. CBB was used as an loading control. **C.** LeY level was detected by immunofluorescent staining. **D.** pEGFR, EGFR, pERK, ERK, pp38 and p38 were detected by Western blot. **E.** pp65, p65, pIKB, IKB, pIKK and IKK were detected by Western blot. Rg3, cells treated with Rg3 (50 μg/ml); Mock, cells transfected with vector; shFUT4, cells transfected with FUT4 shRNA; Anti-LeY, blocking with anti-LeY antibody; AG1478, EGFR inhibitor; BAY11-7082, NF-κB inhibitor. GAPDH was used as an internal control. DAPI was used for nuclear staining (magnification, 400x). The data are presented as the mean ± SEM of three independent experiments.

To study the effects of FUT4 on LeY biosynthesis and EGFR activation, as well as MAPK and NF-κB signal pathways, we analyzed the fucosylation using UEA lectin blot (Figure 5A). LeY level were detected using anti-LeY antibody by Western blot (Figure 5B) and immunofluorescent staining (Figure 5C). The results showed that shFUT4 decreased fucosylation and LeY level in A549 cells. The down-regulation of FUT4 also inhibited the expression and activation of pEGFR, as well as pERK and pp38 in MAPK signal pathway (Figure 5D). Furthermore, shFUT4 transfection decreased the expression and activation of NF-κB signaling proteins, including pp65, pIKB and pIKK. The addition of anti-LeY antibody, EGFR inhibitor (AG1478) or NF-κB inhibitor (BAY11-7082) significantly inhibited the activation of these molecules (Figure 5E), indicating that there is a crosstalk in the EGFR activated MAPK and NF-κB signal pathways. Taken together, these results suggest that down-regulating FUT4 expression decreased LeY biosynthesis which leads to the inhibition of EGFR activation, the downstream MAPK and NF-κB signal pathways in lung cancer cells.

### Rg3 inhibited the tumor growth and EMT in xenograft model and metastasis in tail vein injection model

**Figure 6:**
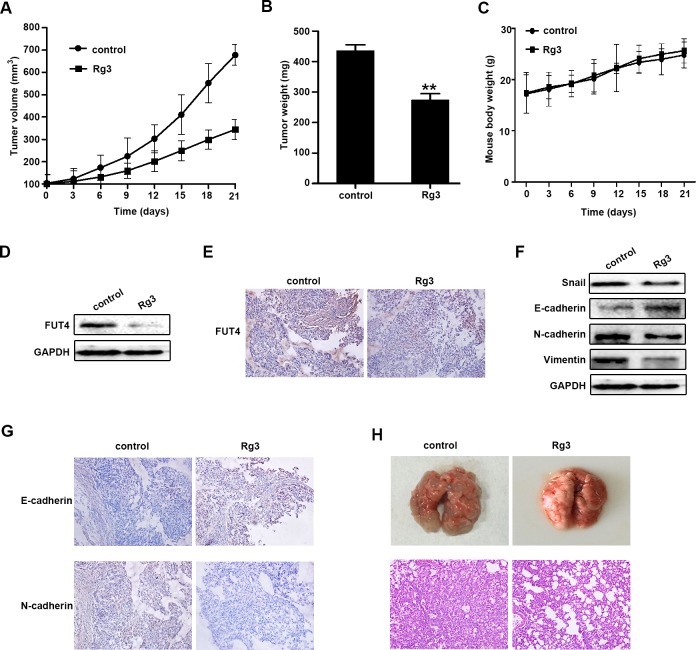
Ginsenoside Rg3 inhibited the growth of NSCLC xenograft tumors, EMT *in vivo* and tumor metastasis in tail vein injection mouse model A549 cells-xenografted nude mice (*n* = 5 per group) were injected with PBS (control) and Rg3 (10 mg/kg body weight) for 21 days. Tumor volume **A.**, tumor weight **B.** and body weight **C.** were presented. FUT4 expression was detected by Western blot **D.** and immunohistochemical staining **E.** in xenograft tumor tissues. Snail, E-cadherin, N-cadherin and Vimentin expression were detected by Western blot **F.**, and immunohistochemical staining of E-cadherin and N-cadherin **G.** in xenograft tumor tissues. GAPDH was used as an internal control (magnification, 200x). **H.** Pictures of mouse lung tissues (upper panel) and lung segments stained with HE (lower panel) in the control and Rg3 treatment groups after tail vein injection for 2 months. HE staining of lung segments (magnification, 200x). The statistical analysis of tumor weight is shown (**, *P* < 0.01). The data are presented as the mean ± SEM of three independent experiments.

To further investigate the inhibition of Rg3 in EMT and metastasis *in vivo*, A549 cells were inoculated into nude mice. The tumor volume and weight were analyzed in the control and Rg3 treatment group. As shown in Figure 6A, 6B, Rg3 treatment significantly reduced tumor volume and weight in xenografts. Rg3 didn't have obvious changes about the body weight of the xenograft mice (Figure 6C). FUT4 expression was examined by Western blot (Figure 6D) and immunohistochemical staining (Figure 6E). The results showed that Rg3 treatment group had a lower expression of FUT4 compared with the untreated control group. EMT marker proteins were examined, and the Rg3 treatment group had a higher expression of E-cadherin and a lower expression of Snail, N-cadherin, and Vimentin by Western blot (Figure 6F), and a similarly stronger staining of E-cadherin and a weaker staining of N-cadherin by immunohistochemical staining (Figure 6G) compared with the control group. These data showed that Rg3 significantly inhibited EMT *in vivo*. To further examine the inhibition of Rg3 in tumor metastasis, A549 cells were injected into mice via the tail vein. Tumor metastasis nodules in Lung tissues of Rg3 group were less and smaller than that control group after treatment for 2 months (Figure 6H).

## DISCUSSION

Currently, the primary therapeutic agents for lung cancer include cisplatin, pemetrexed and erlotinib. Cisplatin exhibits an anti-tumor effect by impairing the structure and function of DNA, whereas the action of pemetrexed is through regulating intracellular folate-dependent metabolism and inhibiting cancer cell replication and proliferation. Erlotinib is an EGFR-targeted drug that inhibits tumor growth by suppressing EGFR and its downstream signal pathways. However, clinical data have shown a limited application of these drugs at tolerated doses due to their toxic side effects and drug resistance [21, 22]. Recent years, a capsule named Shen-Yi, which contains Rg3 as its main ingredient, was approved by the CFDA for cancer therapy, Rg3 is a beneficial adjuvant chemotherapy for lung cancer because it has the capacity to enhance the patients' immune and improve quality of life [23]. Rg3 can enhance the sensitivity to chemotherapy when used in combination with cisplatin or docetaxel [24, 25]. In our study, we investigated the ability of Rg3 to inhibit the EMT of lung cancer cells *in vitro* and *in vivo*. We found that the inhibition of Rg3 in lung cancer EMT was dose- and time-dependent. Moreover, *in vivo*,** our study indicated that Rg3 could suppress the volume and weight of xenograft tumors, and significantly decreased tumor metastasis nodules in lung tissues by tail vein injection. Thus, Rg3 is a potential anti-metastasis agent for lung cancer.

Rg3 is an antitumor drug directed at multiple targets. Rg3 induced the apoptosis of breast cancer cells by inhibiting phorbol ester-induced COX-2 expression and NF-κB activation [26]. Rg3 inhibited the proliferation of melanoma cells by down-regulating HDAC3 and enhancing p53 acetylation [27]. Rg3 suppressed angiogenesis in HIF-1α-mediated acute leukemia by inhibiting PI3K and ERK1/2 signal pathways [28]. To further investigate the mechanism of Rg3 in lung cancer, we detected the expression of FUT4 in the lung cancer tissues, and the inhibitory effect of Rg3 on FUT4 *in vitro* and *in vivo*. We found that FUT4 was highly expressed in lung cancer tissues compared with the normal lung tissues, and Rg3 significantly down-regulated FUT4 expression in a dose- and time-dependent manner. Our study also indicates that down-regulation of FUT4 by Rg3 is correlated to the EMT in lung cancer cells. FHIT suppressed the metastasis of lung cancer by inhibiting micro-RNA-mediated EMT [29], and KY-05009 and TNIK inhibited the invasion of human lung cancer cells by down-regulating TGF-β1-mediated EMT [30]. In our present study, we also found that Rg3 suppressed migration, invasion and EMT in lung cancer cells. These findings suggest that Rg3 is a important inhibitory EMT agent by targeting FUT4 in lung cancer.

**Figure 7:**
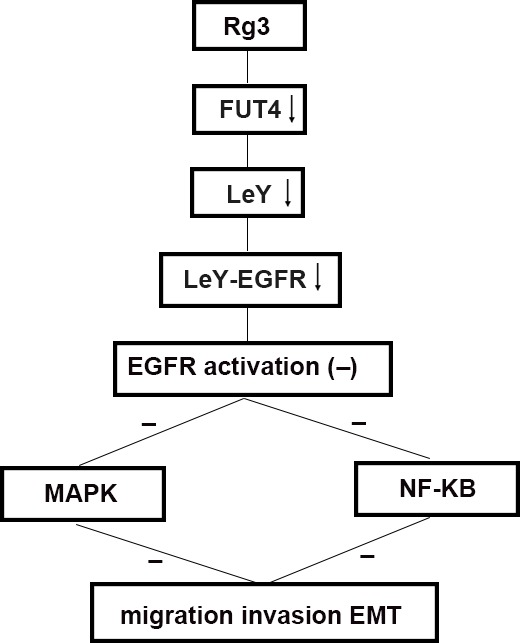
Schematic of the antimetastasis mechanism of Rg3 Schematic to illustrate the hypothetic mechanism that Rg3 down-regulates FUT4 expression, reduces LeY biosynthesis, inhibits EGFR activation by decreasing the biosynthesis of EGFR-carrying LeY, blocks MAPK and NF-κB signal pathways, thus, inhibits migration, invasion and EMT

FUT4 is the key enzyme in the synthesis of LeY. Our previous data confirmed that FUT4 cDNA transfection upregulated FUT4 expression and increased LeY biosynthesis level; whereas down-regulated FUT4 with shFUT4 inhibit LeY biosynthesis [31]. The elevated LeY located on EGFR could induce the activation of EGFR. LeY can induce the activation of EGFR, and promote the migration of cancer cells by the glycosylation of EGFR; whereas anti-LeY antibody inactivated EGFR. In this study, we found that the LeY level and the activation of EGFR (pEGFR) was significantly decreased in shFUT4-transfected A549 cells. EGFR is a critical upstream molecule, and involved in the crosstalk among many signal pathways [32]. Because of the complex of EMT, it is possible that the overlapping and cross-talk pathways are interacted [33]. EGFR activation can induce the both MAPK and NF-κB signal pathways which are involved in EMT. Platycodin D which is an active triterpenoid saponin from Platycodon grandiflorum inhibited migration, invasion, and growth of MDA-MB-231 human breast cancer cells via suppression of EGFR-mediated Akt and MAPK pathways [34]. EGFR/NF-κB signal pathway is correlated with EMT and metastasis. DLC-1 (Loss of deleted in liver cancer-1) induced mitochondrial apoptosis, and inhibited EMT in nasopharyngeal carcinoma by targeting the EGFR/Akt/NF-κB pathway [35]. Scutellarein which is botanical extract bezielle inhibited the proliferation of human lung cancer through ERK and NF-κB mediated by EGFR [36]. We found that down-regulating FUT4 by Rg3 and shFUT4 transfection reduced LeY biosynthesis, inhibited EGFR activation, and blocked the downstream MAPK and NF-κB signal pathways. Therefore, FUT4 can regulate those effectors (FUT4/LeY/LeY-EGFR/MAPK and NF-κB/EMT markers), which triggers EMT occurrence. The mechanistic detail is shown in Figure 7, which helps to state the novel idea of this paper clearly.

Studies have revealed that changes in glycosylation closely correlated with EMT in tumors. For instance, GnT-3 inhibited EMT by changing the glycosylation of E-cadherin, which reduced the phosphorylation of β-catenin [37]. However, GnT-5 promoted EMT by activating EGFR signaling [38]. Overexpression of GALNT14 promoted the occurrence of EMT [39]. In our previous study, we found that FUT4 inactivated GSK3b by inducing PI3K/AKT phosphorylation and further elevated Snail and promoted EMT. However, it was not clear whether FUT4 and EMT are correlated in lung cancer. Our results revealed that both FUT4 and EMT marker N-cadherin were consistently elevated in the lung cancer tissues, indicating there was an intrinsic linkage between them. The further study showed that down-regulating FUT4 increased E-cadherin, and decreased Snail, N-cadherin and Vimentin in lung cancer cells, and thus, hindering EMT. The mechanism exploration of FUT4 in EMT confirmed that the down-regulated FUT4 could decrease LeY biosynthesis, block EGFR-activation, as well as inactivate MAPK and NF-κB signal pathways as shown in Figure 7. FUT4 is an effective target in suppressing lung cancer EMT.

In summary, we found that Rg3 inhibited lung cancer migration and invasion, possibly by inhibiting EMT. In addition, a high expression of FUT4 was closely correlated with EMT in lung cancer. Down-regulation of FUT4 could decrease the expression of EMT marker proteins. These evidence showed that FUT4 could be a potential target for the regulation of EMT, which may be an attractive strategy in inhibiting invasion and metastasis in the treatment of lung cancer. Rg3 could significantly inhibited FUT4 expression. Therefore, we hypothesized that FUT4 is an important target of Rg3′s inhibition EMT in lung cancer. Among these characteristics, Rg3 is an efficient, low-toxicity and multi-target traditional Chinese medicine, and it is expected to become an important agent in the comprehensive treatment of lung cancer, which may improve the current strategy of lung cancer therapy. The molecular mechanism of Rg3′s inhibition in the invasion of lung cancer by targeting EMT, which may be a more effective therapeutic strategy.

## MATERIALS AND METHODS

### Ethics statements

The clinical samples got the approval of the clinical ethics review board in the First Affiliated Hospital of Dalian Medical University. All animal experimental procedures were conducted in conformity with the guidelines for the care and use of laboratory animals in Dalian Medical University.

### Clinical samples and cell lines

All lung cancer paraffin sections and lung cancer tissues were obtained from the First Affiliated Hospital of Dalian Medical University. The lung cancer tissues were kept in liquid nitrogen for protein extraction. Human NSCLC A549, H1299, H358 cell lines were purchased from American Type Culture Collection (ATCC, Manassas, VA). A549, H1299, H358 cells were cultured in RPMI 1640 medium supplemented with 10% fetal bovine serum (TransGen) and 1% penicillin-streptomycin (TransGen) at 37°C in humidified air containing 5% CO_2_.

### Reagents

20 (R)-Rg3 was provided by Dalian Fusheng Pharmaceutical Company (Dalian, China). Rg3 was dissolved in serum-free culture medium, and filtered through a 0.22 mm sterile membrane. The sources of regents were as follows: AG-1478 (Selleck Chemicals, USA); BAY11-7082, EGFR, pEGFR, ERK, pERK, pp38 and p38 (Beyotime); MMP2, MMP9, E-cadherin, FUT4 and GAPDH (Proteintech); Snail, N-cadherin and Vimentin (Bioworld); LeY (Abcam); UEA (LifeSpan BioSciences); p65, pp65, IKB, pIKB, IKK and pIKK (CST). The primers were as follows: Snail, CAGACCCACTCAGATGTCAAGAA(F), GGGCAGGTATGGAGAGGAAGA(R), E-cadherin, CAACGACCCAACCCAAGAA(F), CCGAAGAAACAGCAAGAGCA (R), N-cadherin, AAAGAACGCCAGGCC AAAC(F),GGCATCAGGCTCCACAG T GT(R), Vimentin, CGTCTCTGGCACGTCTTGAC(F), GCTTGGAAACATCCACATCGA(R), GAPDH, GTGTCCGTCGTGGATCTGA(F), GCTTCACCACCTTCTT GATGTCAT (R).

### Construction of RNA interference sequences and transfection

The sequences of FUT4 short hairpin in RNA were as follows: 5′- GATCCGCCTGGCAAGTAACCTCTTCTCAAGAGAAAGAGGTTACTTGCCAGGCTTA - 3′, 5′-AGCTTAAAGCCTGGCAAGTAACCTCTTTCTCTTGAGAAGAGGTTACTTGCCAGGCG - 3′. All sequences were purchased from the GenePharma Company (Shanghai, China). A549 cells were seeded into 6-well plate. When cells reached 60-70% confluence, shFUT4 and vector were transiently transfected into the cells using Lipofectamine 2000 Reagent (Invitrogen) according to manufacturer's instructions. The transfection reagent was removed after 5 h, and the cells were harvested after 48 h.

### Gelatin zymography

Cells were seeded into 6-well plate. The supernatants after the different treatment were collected. The supernatant protein (40μg /lane) was loaded, and electrophoresed on 8% SDS-polyacrylamide gels copolymerized with 1% gelatin. After electrophoresis, the gels were washed five times in 2.5% Triton X-100 (20 min each), and two times in buffer without Triton X-100 to remove Triton X-100, and then incubated in 50 mmol/l Tris-Cl, pH 7.6, and 5 mmol/l CaCl_2_ (18 h, 37°C). The gels were stained with 0.1% Coomassie blue R250 and destained in 10% isopropanol and 10% acetic acid in H_2_O. MMP2 and MMP9 level were detected as transparent bands on the blue background of a Coomassie blue-stained gel.

### Wound-healing assay

Cells were seeded in 6-well plate for 24 h. The monolayers were scratched with a 200 μl pipette tip, and washed with media without serum to remove the detached cells. The wounded areas were observed and imaged under microscope.

### Migration and invasion

Cell migration and invasion were performed in a 24-well plate with 8-mm pore size chamber inserts (Corning, New York, NY, USA). For migration assays, the cells (4×10^4^/well) were placed into the upper chamber with the non-coated membrane. For invasion assays, cells (1×10^5^ /well) were placed into the upper chamber with the Matrigel-coated membrane, which was diluted with serum-free culture medium. In both assays, cells were suspended in 200 μl of RPMI 1640 medium serum-free, and seeded into the upper chamber. In the lower chamber, 800 μl of RPMI 1640 medium supplemented with 10% fetal bovine serum was added. After incubation for 24 h at 37°C and 5% CO_2_, the chambers were fixed with 100% methanol for 20 min, and stained with 0.1% crystal violet for 15 min. Images were captured with an Olympus BX83 fluorescence microscope (Japan).

### Quantitative real-time PCR

Quantitative real-time PCR (qPCR) was used to measure the mRNA expression. Cells were plated into a 6-well plate for 48 h. Total RNA was extracted using TRIzol (Invitrogen) according to the manufacturer's protocol. RNA was reverse transcribed into cDNA using PrimeScriptTMRT reagent kit (Takara, Japan). qPCR reaction was performed in a Thermal Cycler Dice system (Takara, Japan). The PCR program included 45 cycles of 95°C for 1 sec and 60°C for 5 sec. Quantified data were normalized to GAPDH, and the relative quantity was calculated using the 2^−ΔΔCT^ method.

### Western blot

Cells and tumor tissues were lysed. Protein concentration was determined with Coomassie protein assay reagent using bovine serum albumin as a standard. Total protein was run on a 10% SDS-PAGE gel followed by transferring to a nitrocellulose filter membrane (NC), and blocking with 5% non-fat dry milk in TBST for 2 h. The membrane was incubated with the primary antibody overnight followed by incubation with goat anti-rabbit IgG or goat anti-mouse IgM, and HRP-linked antibody for 1 h. An enhanced chemiluminescence (ECL) detection system (Bio-Rad) was used to visualize immunoreactive bands.

### Immunofluorescent staining

After washing with PBS, cells plated on cover slips were fixed with 4% paraformaldehyde for 30 min. Cells were then permeabilized with 0.1% Triton X-100 for 5 min. After being blocked with complete serum for 30 min at 37°C, the cells were incubated with primary antibody at 4°C overnight. The cells were then incubated with FITC-conjugated goat anti-mouse IgM or TRITC-conjugated goat anti-rabbit IgG (Sigma-Aldrich, St. Louis, MO, USA) for 1 h. Images were captured with an Olympus BX83 fluorescence microscope (Japan).

### Xenograft tumor models of human NSCLC

Male nude mice (Balb/c-nu/nu) were obtained from Animal Center (Dalian Medical University). The animals (4-6 weeks of age) were housed in specific pathogen-free (SPF) environment, and provided sterile food and water *ad libitum*. The mice were acclimatized for one week before being used for experiments. A549 cells (2×10^6^) suspended in 200μl of PBS were injected subcutaneously into the right flank of the mice. After one week of the injection, tumor-bearing mice were randomly divided into Rg3 and control groups *(n* = 5 per group). Rg3 (10 mg/kg body weight) was administered to mice at once every three days for 3 weeks via intraperitoneal injection, whereas the control group received PBS only. Tumor volume and the whole body weight were measured every three days after tumor inoculation. The tumor volume was computed according to the following formula: tumor volume (mm^3^) = 1/2 × a (tumor length) × b^2^(tumor width). At the end of the experimental period, all animals were sacrificed by cervical decapitation, the tumor tissues were excised aseptically, and the weight was recorded and used for further study.

### Immunohistochemical staining in xenograft tumors

The excised tumor tissues were fixed in 5% formalin for 24 h before being embedded in paraffin and sectioned (5 μm sections) in a standard manner. The slides were deparaffinized and rehydrated using standard techniques. Non-specific binding sites were blocked with complete serum at 37°C for 30 min. Then, the tissue sections were incubated with the primary antibody at 4°C overnight. The sections were allowed to incubate in a humidified chamber for 1 h at room temperature. The slides were incubated for 10 min at room temperature with a secondary antibody. After blocking the endogenous peroxidase activity with methanol and hydrogen peroxide, the slides were incubated with a streptavidin-peroxidase complex. Diaminobenzidine was used as a chromogen to the protein. The signal was visualized with peroxidase-labeled streptavidin-complexed DAB, and the sections were briefly counterstained with hematoxylin (HE). A yellowish-brown stain indicated a positive result. Images were captured with an Olympus BX51 microscope (Japan).

### Development of tail vein injection lung cancer models

The 4-6 week old male nude mice (Balb/c-nu/nu) were obtained from Animal Center (Dalian Medical University). A549 cells (2×10^6^) suspended in 200μl of PBS were injected into the tail vein of the mice. Rg3 group mice were treated intraperitoneally with Rg3 (10 mg/kg body weight) once every three days for 2 months.

### Statistical analyses

Data were expressed as mean ± SEM of three independent experiments with GraphPad Prism software. The Student's t-test was used to make a statistical comparison between groups. *P* < 0.05 was considered statistically significant.

## SUPPLEMENTARY MATERIAL FIGURES


