# Supplementary material for: Ginsenoside Rg3 inhibits epithelial-mesenchymal transition (EMT) and invasion of lung cancer by down-regulating FUT4
Source: Oncotarget. 2015 Dec 2;7(2):1619–32. doi: 10.18632/oncotarget.6451 (PMC4811485; doi:10.18632/oncotarget.6451)
Supplement: Supplementary file 1 [file oncotarget-07-1619-s001.pdf]

Ginsenoside Rg3 inhibits epithelial-mesenchymal transition (EMT) and invasion of lung cancer by down-regulating FUT4

Supplementary Material

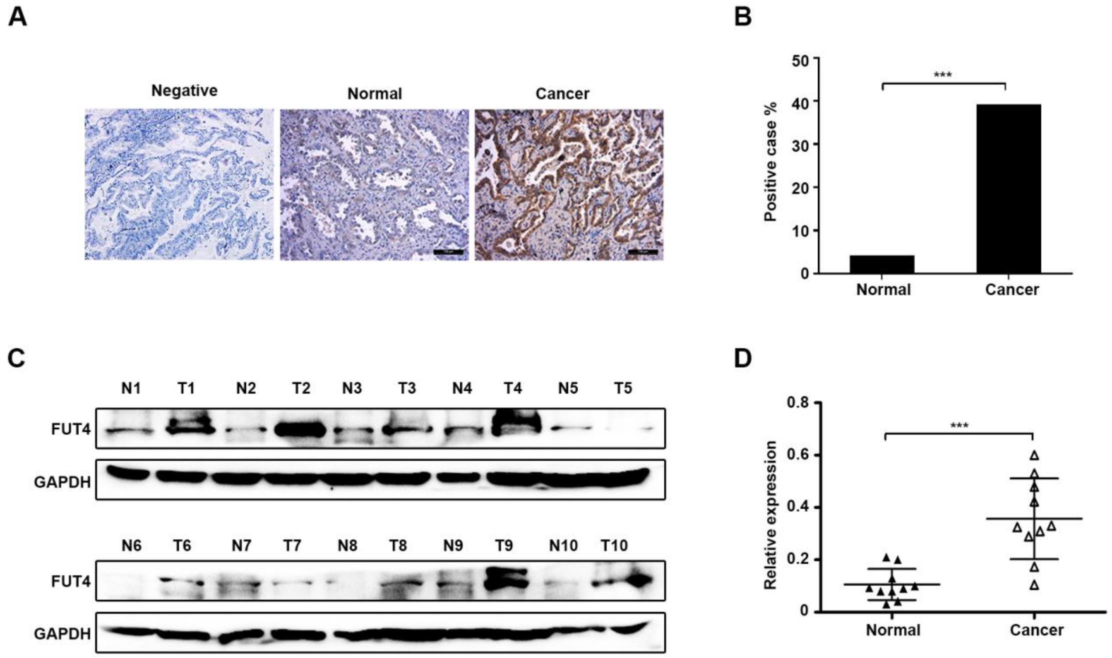

Supp. Figure1: 35 normal lung and 54 lung cancer paraffin sections were collected, and observed FUT4 expression by IHC staining in A. and statistical analysis in B.. The fresh tissues from normal and tumor lung tissues to analyze FUT4 expression by Western blot in C. and statistical analysis in D..

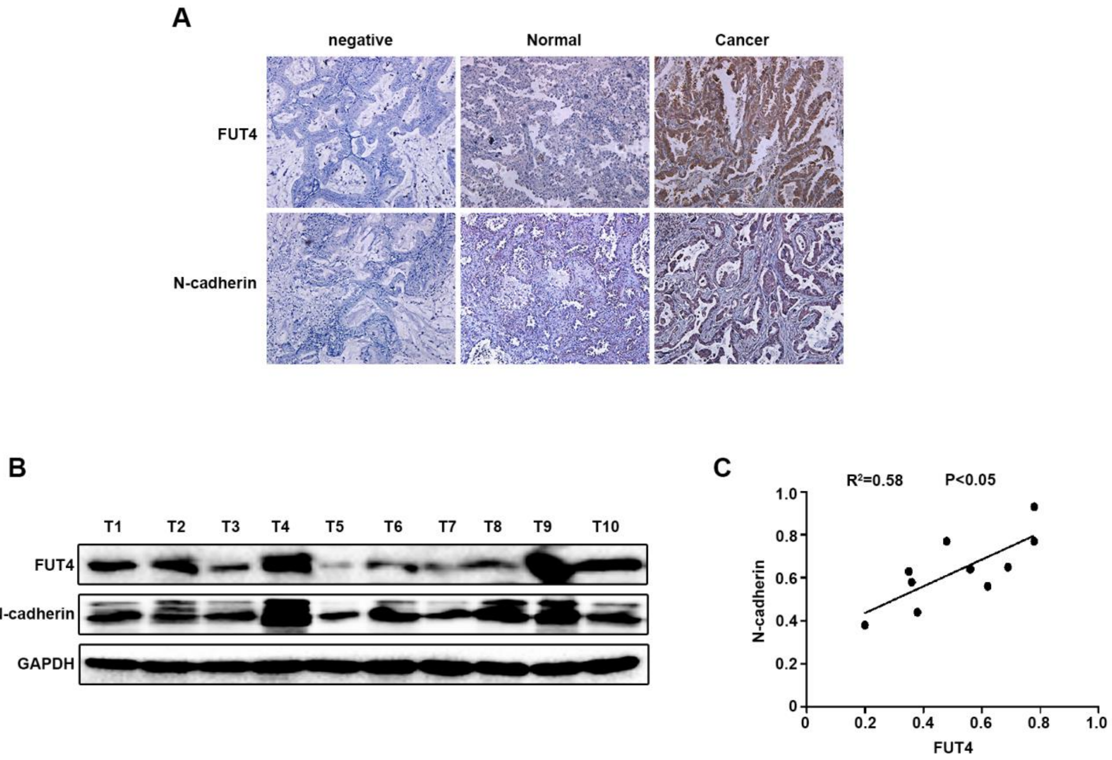

Supp Figure2: The paraffin sections from normal control and lung cancer patients were used to detect FUT4 and N-cadherin expression which is one of the representative genes in EMT by IHC in A. The parallel analysis of FUT4 and N-cadherin expression from lung cancer fresh tissues by Western blot in B. , C..
